# Dectin-1 Stimulation of Hematopoietic Stem and Progenitor Cells Occurs *In Vivo* and Promotes Differentiation Toward Trained Macrophages via an Indirect Cell-Autonomous Mechanism

**DOI:** 10.1128/mBio.00781-20

**Published:** 2020-06-23

**Authors:** Cristina Bono, Alba Martínez, Javier Megías, Daniel Gozalbo, Alberto Yáñez, M. Luisa Gil

**Affiliations:** a Departamento de Microbiología y Ecología, Universitat de València, Burjassot, Spain; b Estructura de Recerca Interdisciplinar en Biotecnologia i Biomedicina (ERI BIOTECMED), Universitat de València, Burjassot, Spain; c Departamento de Patología, Universitat de València, Valencia, Spain; Texas Christian University

**Keywords:** hematopoietic stem and progenitor cells, *Candida albicans*, dectin-1, TLR2, macrophages, trained immunity

## Abstract

Toll-like receptor (TLR) agonists drive hematopoietic stem and progenitor cells (HSPCs) to differentiate along the myeloid lineage. In this study, we used an HSPC transplantation model to investigate the possible direct interaction of β-glucan and its receptor (dectin-1) on HSPCs *in vivo*. Purified HSPCs from bone marrow of B6Ly5.1 mice (CD45.1 alloantigen) were transplanted into dectin-1^−/−^ mice (CD45.2 alloantigen), which were then injected with β-glucan (depleted zymosan). As recipient mouse cells do not recognize the dectin-1 agonist injected, interference by soluble mediators secreted by recipient cells is negligible. Transplanted HSPCs differentiated into macrophages in response to depleted zymosan in the spleens and bone marrow of recipient mice. Functionally, macrophages derived from HSPCs exposed to depleted zymosan *in vivo* produced higher levels of inflammatory cytokines (tumor necrosis factor alpha [TNF-α] and interleukin 6 [IL-6]). These results demonstrate that trained immune responses, already described for monocytes and macrophages, also take place in HSPCs. Using a similar *in vivo* model of HSPC transplantation, we demonstrated that inactivated yeasts of Candida albicans induce differentiation of HSPCs through a dectin-1- and MyD88-dependent pathway. Soluble factors produced following exposure of HSPCs to dectin-1 agonists acted in a paracrine manner to induce myeloid differentiation and to influence the function of macrophages derived from dectin-1-unresponsive or β-glucan-unexposed HSPCs. Finally, we demonstrated that an *in vitro* transient exposure of HSPCs to live C. albicans cells, prior to differentiation, is sufficient to induce a trained phenotype of the macrophages they produce in a dectin-1- and Toll-like receptor 2 (TLR2)-dependent manner.

## Abstract (precis)

Invasive candidiasis is an increasingly frequent cause of serious and often fatal infections. Understanding host defense is essential to design novel therapeutic strategies to boost immune protection against Candida albicans. In this article, we delve into two new concepts that have arisen over the last years: (i) the delivery of myelopoiesis-inducing signals by microbial components directly sensed by hematopoietic stem and progenitor cells (HSPCs) and (ii) the concept of “trained innate immunity” that may also apply to HSPCs. We demonstrate that dectin-1 ligation *in vivo* activates HSPCs and induces their differentiation to trained macrophages by a cell-autonomous indirect mechanism. This points to new mechanisms by which pathogen detection by HSPCs may modulate hematopoiesis in real time to generate myeloid cells better prepared to deal with the infection. Manipulation of this process may help to boost the innate immune response during candidiasis.

## INTRODUCTION

Invasive candidiasis is an increasingly frequent cause of serious and often fatal infections in hospitalized and immunosuppressed patients. Innate immune mechanisms that effectively sense and eliminate fungi are crucial for resistance to candidiasis. During infection, myeloid cells can detect Candida albicans cells through many pattern recognition receptors (PRRs), including different members of the Toll-like receptor (TLR) and C-type lectin receptor (CLR) families, and are responsible for microbial killing, antigen processing and presentation to initiate the adaptive immune response, as well as for releasing proinflammatory cytokines and chemokines to recruit and activate other leukocytes (1, 2).

It has been known for more than a decade that, in addition to mature myeloid cells, murine and human hematopoietic stem and progenitor cells (HSPCs) also express some functional PRRs and that TLR signaling on hematopoietic stem cells (HSCs) provokes cell cycle entry and myeloid differentiation (3–5). This observation suggested that TLRs may play a role in hematopoiesis during infection, as infectious agents accelerate myeloid development to allow for the rapid mobilization of myeloid effector cells in the periphery, a process called emergency myelopoiesis (6).

Our group previously demonstrated that inactivated C. albicans yeasts induce the proliferation of HSPCs and their differentiation toward the myeloid lineage *in vitro*. This response requires signaling through TLR2 and dectin-1 and gives rise to functional phagocytes that are able to internalize yeasts and secrete proinflammatory cytokines (7–9). Moreover, using an *in vitro* model of HSPC differentiation, we have shown that detection of microorganism-associated molecular patterns (MAMPs) by HSPCs impacts the antimicrobial function of the macrophages they produce (10). Pure soluble TLR2 and TLR4 ligands generate macrophages with a diminished ability to produce inflammatory cytokines (tolerized macrophages), whereas HSPC activation in response to C. albicans or dectin-1 ligands leads to the generation of macrophages that produce higher levels of cytokines (trained macrophages) than control macrophage colony-stimulating factor (M-CSF)-derived macrophages (11, 12). All these *in vitro* results indicate that PRR-mediated recognition of C. albicans by HSPCs may help to replenish the innate immune system and to generate trained myeloid cells to deal with the pathogen during an infection.

In addition, these newly described mechanisms have been explored in some *in vivo* models. Using an experimental model of HSPC transplantation (from wild-type mice into TLR2 or TLR4 knockout mice, which were then injected with soluble TLR2 or TLR4 ligands, respectively) we have shown that HSPCs are directly stimulated by TLR agonists *in vivo*, inducing differentiation toward macrophages. As recipient mouse cells do not recognize the pure TLR ligands injected, interference by soluble mediators secreted by recipient cells is negligible (13). Furthermore, in this transplantation model, macrophages derived *in vivo* from HSPCs exposed to the TLR2 agonist Pam_3_CSK_4_, exhibited reduced production of inflammatory cytokines (10).

Despite having known that TLRs induce HSPC differentiation toward macrophages for more than a decade, the molecular mechanisms involved have not yet been completely elucidated (6, 14). Although cytokines indirectly produced by HSPCs, such as interleukin 6 (IL-6) have been demonstrated to act in an autocrine/paracrine manner to induce myeloid development (15), it is unclear whether TLR signaling initiates myeloid differentiation directly, in a cell-intrinsic manner (16–19).

In this study, we have extended our previous *in vivo* studies of HSPC transplantation to demonstrate the role of dectin-1 signaling in HSPC differentiation and generation of trained macrophages. Moreover, using an *in vitro* model of coculture, we have studied the possible direct or indirect mechanisms by which TLR2 or dectin-1 induces HSPC differentiation and confers a tolerized or trained phenotype, respectively, to the mature myeloid cells they generate. Our work shows that macrophage differentiation can be directly induced by TLR2 signaling. However, the tolerized phenotype and the dectin-1-mediated differentiation to trained macrophages are mostly produced by indirect mechanisms. Finally, we demonstrate that a transient exposure of HSPCs to live C. albicans cells, prior to differentiation, is sufficient to induce a trained phenotype for the macrophages they produce in a dectin-1- and TLR2-dependent manner. Taken together, these data indicate that HSPCs can sense C. albicans directly during an infection to rapidly generate trained macrophages to deal with the pathogen.

## RESULTS

### Transplanted CD45.1 Lin^−^ cells in dectin-1^−/−^ CD45.2 mice respond to the dectin-1 ligand and are directed to produce macrophages.

**FIG 1:**
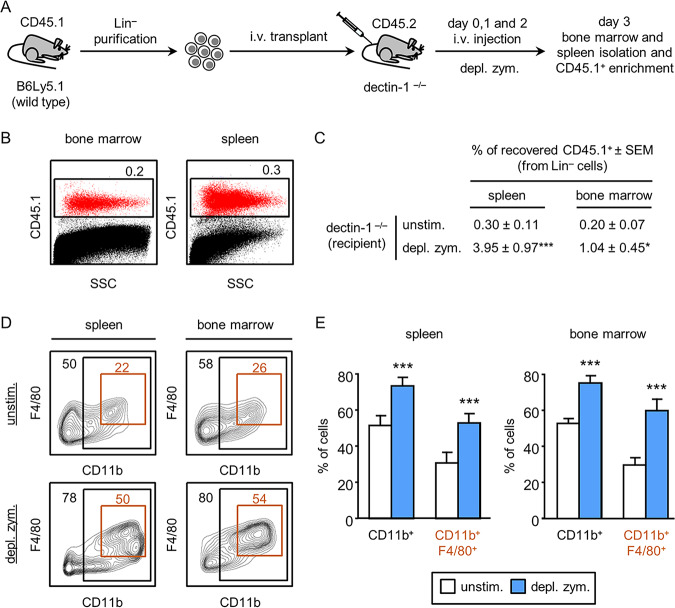
*In vivo* differentiation of transplanted CD45.1 Lin^−^ progenitor cells in response to dectin-1 ligand. (A) Schematic protocol of cell transplantation and stimulation (see Materials and Methods). (B) Three days after transplantation, donor-derived CD45.1 cells were detected in the bone marrow and spleens of CD45.2 control mice (unchallenged with depleted zymosan). Dot plots show side scatter (SSC) against CD45.1 expression of the purified bone marrow and spleen cells. Percentage of recovered CD45.1 cells is calculated as follows: total number of recovered cells × 100/(total number of transplanted cells), where the total number of recovered cells is the percentage of CD45.1 cells determined by flow cytometry × total number of purified cells from spleen or bone marrow/100. Indicated percentages are the means ± SDs from six mice. (C) Percentages of recovered CD45.1 cells from the spleens and bone marrow of dectin-1^−/−^ knockout mice transplanted with CD45.1 Lin^−^ progenitor cells and stimulated daily with 100 μg/day of depleted zymosan, for 3 days. Data represent means ± standard errors of the means (SEMs) from two independent experiments (three mice per condition and experiment). *, *P* < 0.05; ***, *P* < 0.001 with respect to CD45.1 cells recovered from transplanted unstimulated control mice. (D and E) The CD45.1 population was gated, shown in CD11b versus F4/80 contour plots, and subgated as CD11b^+^ and double-positive CD11b^+^ F4/80^+^ cells. The indicated percentages refer to total CD45.1 cells analyzed. Representative plots (D) and bar graphs (E) of data expressed as means ± SDs from two independent experiments (three mice per condition and experiment). ***, *P* < 0.001 with respect to CD45.1 cells recovered from transplanted unstimulated control mice.

Direct *in vivo* interaction between microbial pathogens, or their ligands, and PRRs on HSPCs is difficult to demonstrate, as HSPCs could respond to other stimuli generated when mature immune or nonimmune cells detect microbial products via their PRRs. To investigate the possible direct interaction of β-glucan with dectin-1 on HSPCs *in vivo*, we designed the experimental approach described in Fig. 1A. We purified HSPCs (lineage-marker-negative [Lin**^−^**] cells) from bone marrow of B6Ly5.1 mice (CD45.1 alloantigen) and transplanted these cells into dectin-1^−/−^ mice (CD45.2 alloantigen). We then injected the mice with one dose of depleted zymosan (a pure dectin-1-activating Saccharomyces cerevisiae cell wall preparation of β-glucan) daily for 3 days. Using this experimental approach, the recipient mouse cells do not recognize the ligand injected, and so there should not be cytokines or soluble mediators secreted by recipient cells. At day three, bone marrow and spleen cells were depleted of CD45.2 recipient cells for the enrichment of CD45.1 donor cells and analyzed by flow cytometry. Approximately 0.3% of the transplanted cells were recovered from the spleens and 0.2% were recovered from the bone marrow of unstimulated mice (Fig. 1B). A significant increase in CD45.1 cells was detected in the spleens and bone marrow of dectin-1^−/−^ mice transplanted with Lin**^−^** cells following depleted zymosan challenge (Fig. 1C). These results indicate that dectin-1 signaling resulting from direct detection of depleted zymosan by the transplanted Lin**^−^** cells induces their proliferation and/or improves their survival *in vivo.*

Next, the expression of myeloid (CD11b) and macrophage (F4/80) markers on CD45.1 cells was analyzed. We found that in unstimulated mice, transplanted cells differentiated toward the myeloid lineage, as significant percentages of CD11b^+^ cells (roughly 50%, both in spleen and bone marrow) were detected (Fig. 1D and E). Some of these cells expressed the macrophage marker F4/80 (roughly 25%, both in spleen and bone marrow). After the injection of depleted zymosan into the dectin-1^−/−^ mice, a significant increase among CD45.1 cells of CD11b^+^ (roughly 80%) and CD11b and F4/80 double-positive cells (roughly 52%) was detected, both in spleen and bone marrow. These results demonstrate that HSPCs are directly stimulated by the dectin-1 agonist *in vivo* and that the engagement of this receptor promotes macrophage differentiation.

### Transplanted CD45.2 Lin^−^ cells respond to inactivated C. albicans yeasts and are directed to produce macrophages by dectin-1 and MyD88-dependent signaling.

**FIG 2:**
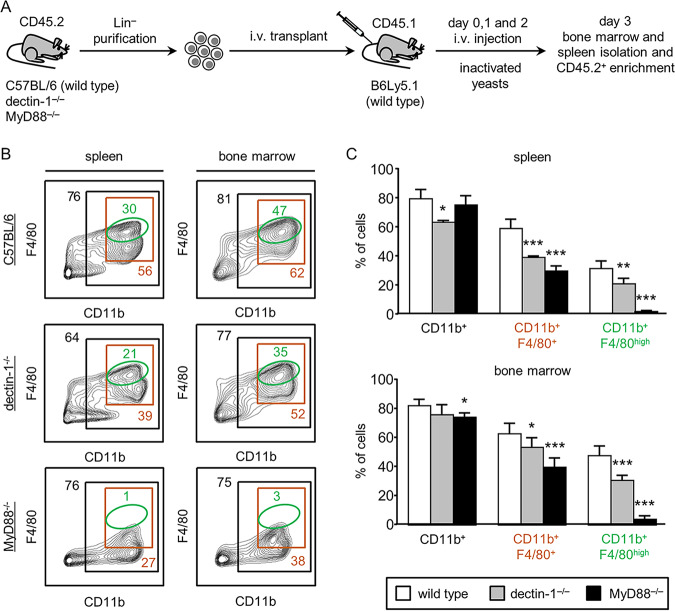
*In vivo* differentiation of dectin-1^−/−^ or MyD88^−/−^ CD45.2 Lin^−^ progenitor cells in response to stimulation with C. albicans inactivated yeasts. (A) Schematic protocol of cell transplantation and stimulation (see Materials and Methods). (B and C) The CD45.2 population was gated, shown in CD11b versus F4/80 contour plots, and subgated as CD11b^+^, double-positive CD11b^+^ F4/80^+^ cells, and CD11b^+^ F4/80^high^ cells. The indicated percentages refer to total CD45.2 cells analyzed. Representative plots (B) and bar graphs (C) of data expressed as means ± SD from two independent experiments (three mice per condition and experiment). *, *P* < 0.05; **, *P* < 0.01; ***, *P* < 0.001 with respect to CD45.2 cells recovered from WT C57BL/6 transplanted progenitor cells.

To investigate the *in vivo* differentiation of transplanted HSPCs in response to C. albicans, Lin^−^ cells were purified from the bone marrow of C57BL/6 mice (CD45.2 alloantigen) and transplanted into B6Ly5.1 mice (CD45.1 alloantigen), which were then injected with one daily dose of 10 × 10^6^ inactivated yeasts for 3 days. Three days after transplantation, bone marrow and spleen cells were enriched for CD45.2 cells by depletion of CD45.1 cells and analyzed by flow cytometry (Fig. 2A). After C. albicans injection, donor HSPCs differentiated into CD11b^+^ cells (roughly 80%), CD11b and F4/80 double-positive cells were roughly 60%, and within the double-positive cells, we detected a subpopulation that expressed higher levels of F4/80 (CD11b^+^ F4/80^high^), which represented 30% of the population in the spleen and 47% in the bone marrow. These data prompted us to investigate whether dectin-1 and/or TLR signaling is playing a role in this HSPC differentiation in response to yeasts. To do this, Lin**^−^** cells from bone marrow of dectin-1^−/−^ or MyD88^−/−^ mice (CD45.2 alloantigen) were transplanted into B6Ly5.1 mice (CD45.1 alloantigen), which were then injected with inactivated yeasts and analyzed as described above (Fig. 2A). Using this experimental approach, the possible differences in CD45.2 cells derived from dectin-1^−/−^ and MyD88^−/−^ mice, in comparison with cells from C57BL/6 control mice, should only be due to defective dectin-1 or TLR signaling in transplanted progenitor cells. The percentages of double-positive CD11b and F4/80 macrophages generated from dectin-1^−/−^ and MyD88^−/−^ progenitors were significantly decreased in comparison to the percentage of macrophages generated from wild-type Lin**^−^** cells (Fig. 2B and C). This decrease was more pronounced in the CD11b^+^ F4/80^high^ subpopulation, especially in mice transplanted with MyD88^−/−^ cells. Dectin-1^−/−^ HSPCs gave rise to 21% and 35% of this macrophage subset in the spleen and bone marrow, respectively, whereas MyD88^−/−^ HSPCs only yielded 1% in the spleen and 3% in the bone marrow. It should be noted that the percentages of total CD11b^+^ cells were quite similar in all cases (except for a slight decrease in the spleens of mice transplanted with dectin-1^−/−^ cells and in the bone marrow of mice transplanted with MyD88^−/−^ cells), suggesting that signaling through dectin-1 and TLRs in response to C. albicans induces differentiation specifically of F4/80-positive macrophages.

Overall, these results suggest that the C. albicans-induced *in vivo* differentiation of Lin**^−^** cells to macrophages is dependent on both TLRs/MyD88 and dectin-1, although the role of TLR signaling seems to be more significant.

### HSPC-derived macrophages by dectin-1 agonist treatment *in vivo* produce higher levels of cytokines than control macrophages.

**FIG 3:**
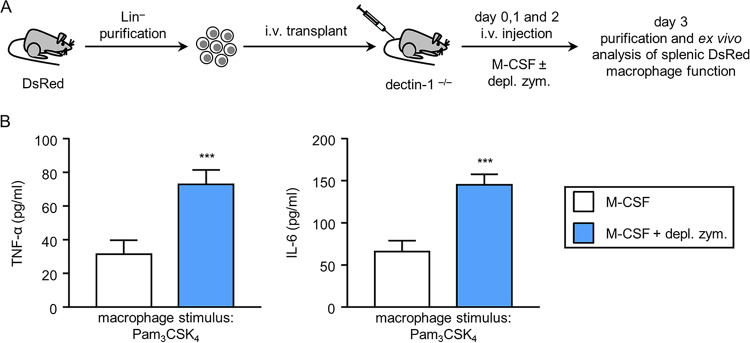
Effect of exposure to depleted zymosan during *in vivo* differentiation of Lin^−^ progenitor cells on macrophage cytokine response. (A) Schematic protocol of cell transplantation and stimulation (see Materials and Methods). (B) Macrophages were stimulated with 100 ng/ml of Pam_3_CSK_4_, and TNF-α and IL-6 levels in 24-h culture supernatants were assessed by ELISA. Triplicate samples were analyzed in each assay. Results are expressed as means ± SDs of pooled data from two experiments. ***, *P* < 0.001 with respect to cytokine production by macrophages derived from Lin**^−^** cells differentiated with M-CSF only, in the absence of depleted zymosan.

We previously showed that *in vitro* HSPC activation in response to C. albicans or depleted zymosan leads to the generation of macrophages that produce higher levels of cytokines (trained macrophages) than control M-CSF-derived macrophages (11). Here, we investigated the function of macrophages produced in response to the detection of depleted zymosan by HSPCs *in vivo*. For this, we used Lin**^−^** cells from DsRed mice to distinguish donor from recipient cells. DsRed HSPCs transplanted into dectin-1^−/−^ mice were induced to differentiate into macrophages by injection of M-CSF in order to permit functional comparisons with control macrophages generated *in vivo*, with or without depleted zymosan (Fig. 3A). Three days after transplantation, spleen cells were enriched for myeloid cells by depletion of lymphocytes and stained for CD11b and F4/80; DsRed double-positive cells were sorted by flow cytometry. *Ex vivo* stimulation of equal numbers of donor-derived macrophages with Pam_3_CSK_4_ revealed that macrophages derived in the presence of depleted zymosan produced higher levels of the inflammatory cytokines tumor necrosis factor alpha (TNF-α) and IL-6 (Fig. 3B). Since the recipient mice are unable to respond to depleted zymosan, this effect must be due to direct detection of the dectin-1 ligand by the donor HSPCs and possibly their progeny. This result demonstrates that HSPCs are stimulated by dectin-1 *in vivo* and are subsequently directed to produce trained macrophages by a cell-autonomous mechanism.

### TLR2 signaling drives direct cell-intrinsic myeloid differentiation from Lin^−^ cells, whereas dectin-1 drives indirect myeloid differentiation.

**FIG 4:**
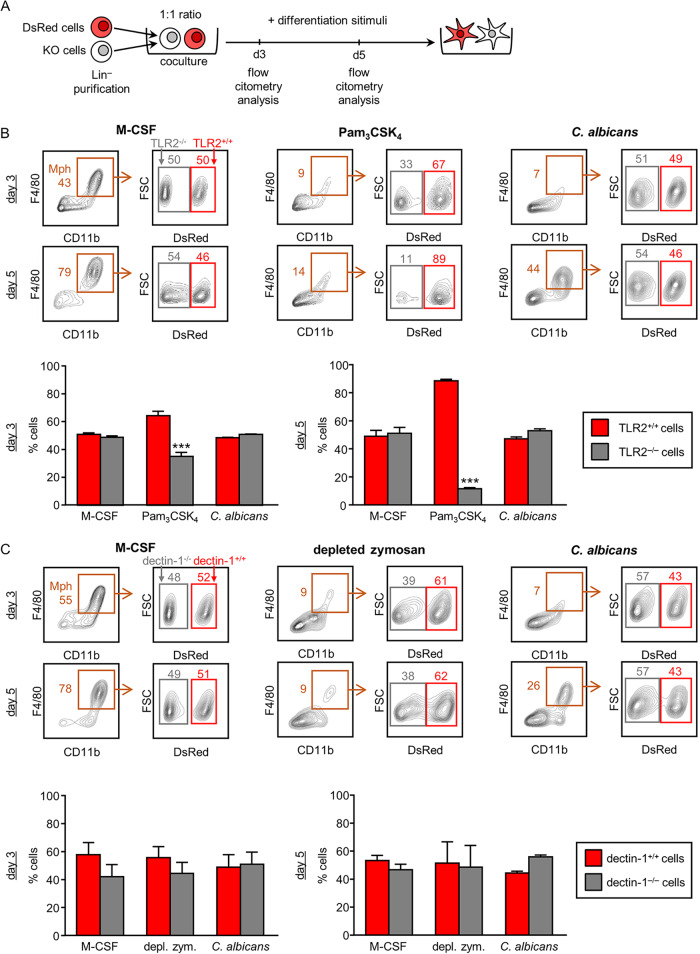
Flow cytometry analyses of Lin**^−^** cells differentiated *in vitro* in response to M-CSF, Pam_3_CSK_4_, depleted zymosan, or C. albicans. (A) Schematic protocol of cell differentiation and assay (see Materials and Methods). DsRed and TLR2^−/−^ Lin**^−^** cells (B) or DsRed and dectin-1^−/−^ Lin**^−^** cells (C) were cultured at a 1:1 ratio and stimulated with M-CSF, inactivated yeasts of C. albicans, or Pam_3_CSK_4_ (B) or depleted zymosan (C) for 3 or 5 days. Cells were labeled with anti-CD11b and anti-F4/80 antibodies and analyzed by flow cytometry. Macrophages (Mph) were gated as double-positive CD11b^+^ F4/80^+^ cells, shown in forward scatter (FSC) versus DsRed contour plots, and subgated as DsRed^+^ and DsRed^−^. The indicated percentages on dot plots refer to total analyzed cells. Bar graphs show means ± SDs of pooled data from 3 independent experiments. ***, *P* < 0.001 with respect to macrophages generated from DsRed Lin**^−^** cells.

To understand whether the mechanisms that drive HSPC differentiation to mature myeloid cells in response to TLR2 and dectin-1 ligands are direct or indirect, we performed differentiation experiments with mixed cocultures of wild-type (WT) and knockout (KO) HSPCs. Lin**^−^** cells were purified from DsRed and TLR2^−/−^ mice and were cocultured at a 1:1 ratio in the presence of M-CSF (as a control for macrophage differentiation), TLR2 ligand (Pam_3_CSK_4_), or inactivated yeasts of C. albicans. The percentage of DsRed-positive (TLR2^+/+^) and -negative (TLR2^−/−^) cells of the differentiated cells (CD11b^+^ and F4/80^+^) was analyzed by flow cytometry after 3 and 5 days of culture (Fig. 4A). As expected, TLR2^−/−^ and TLR2^+/+^ HSPCs produced equivalent numbers of CD11b^+^ F4/80^+^ cells in the M-CSF-stimulated cocultures. However, in response to Pam_3_CSK_4_, TLR2^+/+^ HSPCs produced higher numbers of CD11b^+^ F4/80^+^ cells than TLR2^−/−^ HSPCs (67% TLR2^+/+^ and 33% TLR2^−/−^ at day 3, and 89% TLR2^+/+^ and 11% TLR2^−/−^ at day 5). The lower percentage of differentiated TLR2^−/−^ cells than TLR2^+/+^ cells indicates that TLR2 stimulation of Lin**^−^** cells promotes cell-autonomous direct myeloid differentiation, since the differentiation mediated by indirect mechanisms (caused by molecules released in response to the ligand which could act in an autocrine and/or paracrine way) would be acting in both progenitor genotypes under these culture conditions. In contrast, the percentages of TLR2^+/+^ and TLR2^−/−^ cells of the CD11b^+^ and F4/80^+^ cells were similar in response to inactivated yeasts of C. albicans, indicating that the secretion of soluble mediators was sufficient to elicit myeloid differentiation in the absence of a concurrent TLR2 signal. Moreover, considering that inactivated C. albicans signals via both TLR2 and dectin-1, there could be compensation mechanisms in single-knockout cells leading to the resulting similar macrophage levels observed.

We also performed the same experiment coculturing Lin**^−^** cells from DsRed and from dectin-1^−/−^ mice in the presence of M-CSF, depleted zymosan, or inactivated yeasts of C. albicans (Fig. 4B). The percentages of dectin-1^+/+^ and dectin-1^−/−^ cells of the CD11b^+^ and F4/80^+^ cells were similar in response to all stimuli used: M-CSF, depleted zymosan (not statistically significant for the slight differences observed), and inactivated yeasts.

Overall, these results demonstrate that direct sensing of TLR2 ligands promotes myelopoiesis directly, whereas dectin-1 signaling induces differentiation of HSPCs by indirect mechanisms.

### The tolerized or trained phenotype of macrophages derived from HSPCs treated with TLR2 or dectin-1 agonists, respectively, is mainly conferred by indirect mechanisms.

**FIG 5:**
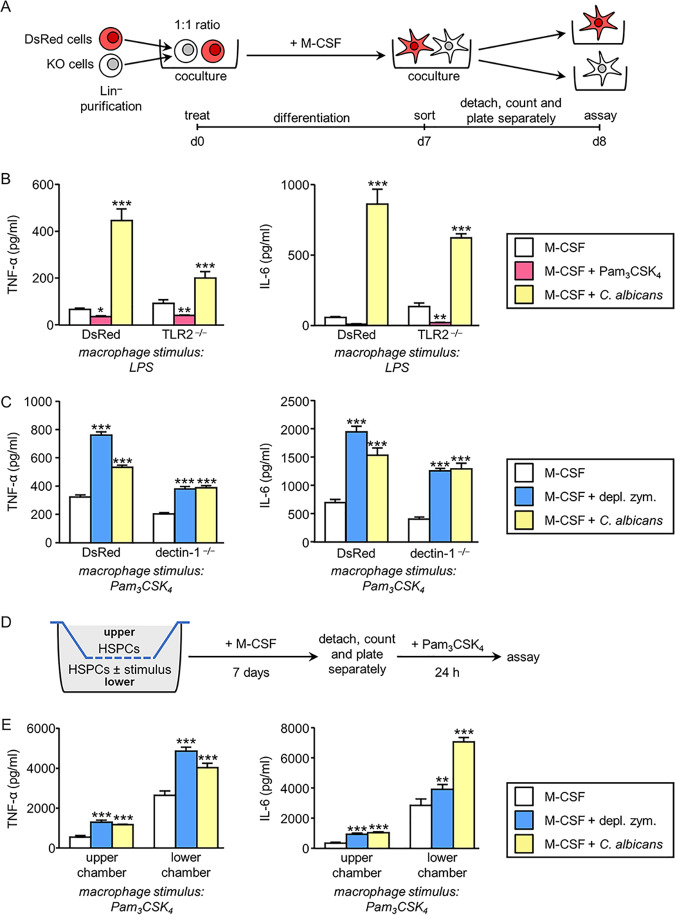
Responsiveness of macrophages derived from Lin**^−^** cells differentiated *in vitro* in response to M-CSF ± stimulus. (A) Schematic protocol of *in vitro* Lin^−^ cell differentiation and sorting (see Materials and Methods). DsRed and TLR2^−/−^ Lin**^−^** cells (B) or DsRed and dectin-1^−/−^ Lin**^−^** cells (C) were cultured at a 1:1 ratio and stimulated with M-CSF in the absence or presence of inactivated yeasts of C. albicans or Pam_3_CSK_4_ (B) or depleted zymosan (C) for 7 days. Cells were labeled with anti-CD11b and anti-F4/80 antibodies, and DsRed-positive and -negative macrophages (CD11b^+^ F4/80^+^) were sorted by flow cytometry and plated separately. (B and C) Macrophages were stimulated with LPS (100 ng/ml) or Pam_3_CSK_4_ (100 ng/ml), and TNF-α and IL-6 levels in 24-h culture supernatants were assessed by ELISA. Triplicate samples were analyzed in each assay. Results are expressed as means ± SD of pooled data from three experiments. *, *P* < 0.05; **, *P* < 0.01; ***, *P* < 0.001 with respect to cytokine production by macrophages derived from Lin**^−^** cells differentiated with M-CSF only, in the absence of additional stimuli. (D) Schematic protocol of *in vitro* Lin^−^ cell differentiation in transwell assays (see Materials and Methods). (E) Macrophages from the upper or lower chamber were separately stimulated with Pam_3_CSK_4_ (100 ng/ml), and TNF-α and IL-6 levels in 24-h culture supernatants were assessed by ELISA. Triplicate samples were analyzed in each assay. Results are expressed as means ± SDs of pooled data from three experiments. **, *P* < 0.01; ***, *P* < 0.001 with respect to cytokine production by macrophages derived from Lin**^−^** cells differentiated with M-CSF, only in the absence of additional stimuli in the lower chamber.

We previously demonstrated that HSPCs exposed to the TLR2 ligand produce soluble factors that act in a paracrine manner on unexposed HSPCs which then generate tolerized macrophages (10). These results prompted us to investigate the mechanisms by which trained macrophages are generated from HSPCs as a consequence of depleted zymosan and C. albicans yeast exposure. To address this, Lin**^−^** cells purified from the bone marrow of DsRed and TLR2^−/−^ or dectin-1^−/−^ mice were cocultured at a 1:1 ratio in the presence of M-CSF and in the presence or absence of Pam_3_CSK_4_, depleted zymosan, or inactivated yeasts of C. albicans. After 7 days, DsRed-positive and -negative macrophages (CD11b^+^ F4/80^+^) were sorted by flow cytometry, plated separately, and stimulated to assess cytokine production (Fig. 5A). The tolerized or trained phenotype was determined using the DsRed macrophages (WT) as controls, which were tolerized in the Pam_3_CSK_4_ cocultures and trained in the depleted zymosan or C. albicans yeast cocultures in comparison to those in the M-CSF cultures.

According to previous results (10), TLR2^−/−^ macrophages derived in the presence of Pam_3_CSK_4_-activated WT cells produced less TNF-α and IL-6 than TLR2^−/−^ macrophages derived with M-CSF in the absence of the TLR2 ligand (Fig. 5B), confirming that soluble factors produced by HSPCs in response to the TLR2 ligand act in a paracrine manner on unresponsive HSPCs (TLR2^−/−^). On the other hand, dectin-1^−/−^ macrophages derived in the presence of depleted zymosan-activated WT cells produced more TNF-α and IL-6 than dectin-1^−/−^ macrophages derived with M-CSF in the absence of depleted zymosan (Fig. 5C), clearly indicating that soluble factors produced by HSPCs in response to dectin-1 ligand act in a paracrine manner on unresponsive HSPCs (dectin-1^−/−^) that then generate trained macrophages. The same effect was observed when TLR2^−/−^ or dectin-1^−/−^ HSPCs were cocultured with WT cells in the presence of C. albicans yeasts (Fig. 5B and C), although, in this model, training in knockout cells in response to yeasts could also be due to alternative PRR stimulation in addition to indirect mechanisms.

Finally, we performed transwell assays, as indicated in Fig. 5D, to determine whether cell contact is important for this effect. We found that macrophages derived from unexposed HSPCs cultured with, but physically separated (upper chamber) from, depleted zymosan- or C. albicans-exposed HSPCs (lower chamber) still exhibited increased TNF-α and IL-6 production (Fig. 5E), indicating that soluble factors produced by the exposed cells confer functional programming (without cell contact) to unexposed cells at some point during their differentiation.

Taken together, these results show that detection of pathogen-associated molecular patterns (PAMPs), including yeasts, by HSPCs defines their secretome, which acts in a paracrine manner and impacts the function of the macrophages they produce.

### Transient exposure of HSPCs to live cells of C. albicans confers a trained phenotype to the macrophages they produce in a dectin-1- and TLR2-dependent manner.

**FIG 6:**
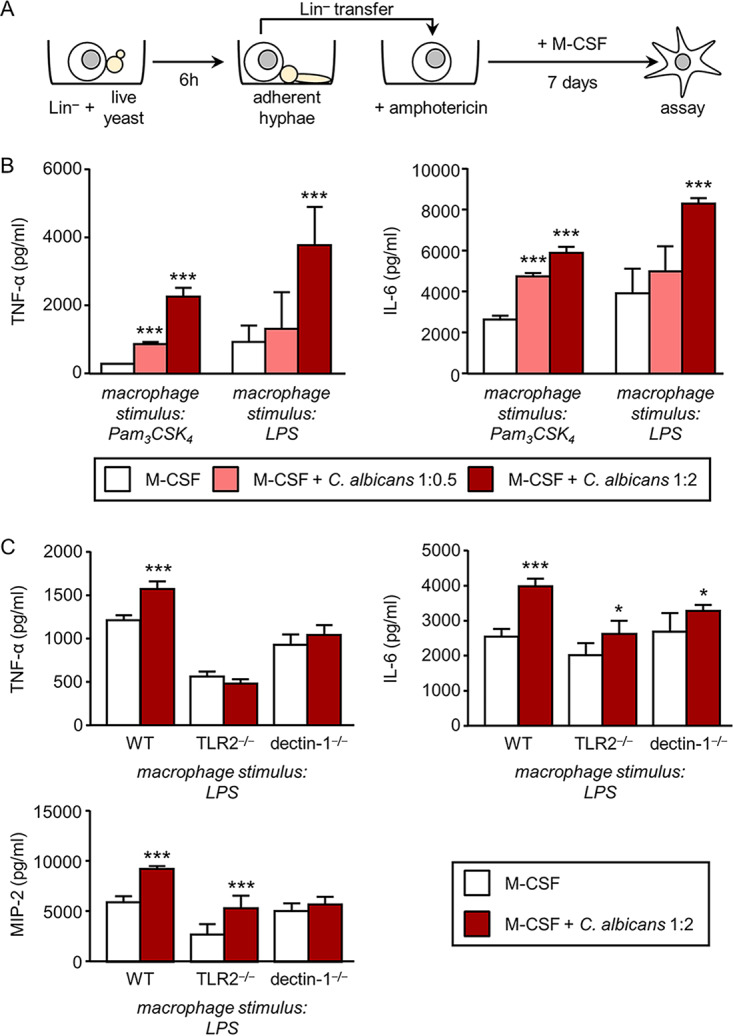
Effect of transient exposure of Lin^−^ progenitor cells to live C. albicans cells prior to differentiation on macrophage cytokine production. (A) Schematic protocol of *in vitro* Lin^−^ cell differentiation and stimulation. Lin**^−^** cells were cultured in the presence or absence of live C. albicans cells at 1:0.5 or 1:2 ratio (progenitor/yeast) for 6 h and then amphotericin B (0.5 μg/ml) was added. Lin**^−^** cells were transferred to a new plate and cultured with M-CSF for 7 days to obtain macrophages. (B) Macrophages were stimulated with 100 ng/ml of Pam_3_CSK_4_, and TNF-α and IL-6 levels in 24-h culture supernatants were assessed by ELISA. (C) Lin**^−^** cells from WT, TLR2^−/−^, or dectin-1^−/−^ mice were cultured in the presence or absence of live C. albicans cells at 1:2 ratio (progenitor/yeast) for 6 h and differentiated as indicated in the schematic protocol in panel A. (C) Macrophages were stimulated, and TNF-α, IL-6, and MIP-2 production was measured as indicated for panel B. Triplicate samples were analyzed in each assay. Results are expressed as means ± SDs of pooled data from two experiments. *, *P* < 0.05; ***, *P* < 0.001 with respect to cytokine production by macrophages derived from Lin**^−^** cells unexposed to C. albicans.

Our previous studies show that *in vitro* and *in vivo* exposure of HSPCs to dectin-1 agonists (depleted zymosan or inactivated yeasts of C. albicans) leads to the generation of trained macrophages (11, 12). But the consequences of exposing HSPCs to inactivated yeasts may be different from those of exposing them to live yeasts, which can express several virulence factors, including the yeast-to-hypha transition and candidalysin secretion (20). Therefore, we next investigated the functional consequences of dectin-1 activation by live C. albicans cells, exclusively at the HSPC stage, prior to macrophage differentiation. For these experiments, Lin**^−^** cells were cultured in the presence or absence of live C. albicans cells at a 1:0.5 or 1:2 ratio (progenitor/yeast) for 6 h. Under these experimental conditions, i.e., a low number of fungal cells, presence of serum, and at 37°C, most of the yeasts performed the conversion from budding yeast to the filamentous growth form. After 6 h of coculture, amphotericin B was added to stop fungal growth. Taking advantage of the high adhesion of hyphae to plastic, Lin**^−^** cells were transferred to a new plate leaving the hyphae adhered to the original plate and were cultured with M-CSF for 7 days to obtain macrophages. Equal numbers of macrophages, derived from unexposed and live C. albicans-exposed HSPCs, were plated to examine their function (Fig. 6A). Results show that the production of TNF-α and IL-6 in response to Pam_3_CSK_4_ or lipopolysaccharide (LPS) is significantly increased in macrophages derived from HSPCs exposed transiently to live C. albicans cells, and that this trained phenotype is dose dependent (Fig. 6B).

Lin**^−^** cells from TLR2^−/−^ or dectin-1^−/−^ mice transiently exposed to live C. albicans cells give rise to macrophages that produce similar levels of TNF-α as macrophages generated from unexposed HSPCs. Although the production of IL-6 was slightly increased in comparison to that in control macrophages, the relative increase (exposed/unexposed) for KO cells was lower (roughly 27%) than the increase for wild-type macrophages (60%). Finally, the production of the chemokine macrophage inflammatory protein 2 (MIP-2) was dependent on dectin-1 but independent of TLR2 (Fig. 6C). These results indicate that in this model of transient exposure to live C. albicans cells, the generation of trained macrophages is partially dependent on both TLR2 and dectin-1. Overall, these results demonstrate that HSPCs can sense live C. albicans cells directly during infection to rapidly generate trained macrophages.

## DISCUSSION

Under physiological conditions, blood cell production is tightly controlled by HSC self-renewal and by their conversion into lineage-committed progenitors. Many cytokines and transcription factors “fine-tune” the proliferation of HSPCs and their differentiation into mature myeloid and lymphoid cells (21). However, accumulated evidence has shown that hematopoiesis is altered during infection, whereby the production of phagocytes, particularly granulocytes and monocytes, becomes predominant with inhibition of other lineage (lymphoid and erythroid) development, a process known as emergency myelopoiesis (14).

The mechanisms by which different pathogen signals are detected and subsequently translated into demand-adapted myelopoiesis are beginning to be understood (14). Moreover, new perspectives on emergency myelopoiesis came when several reports demonstrated that HSPCs express functional PRRs and that PRR signals provoke myeloid differentiation (6). In conventional *in vivo* models of infection, the alterations in hematopoiesis and HSPC populations can be explained by at least two types of mechanisms: (i) MAMPs may directly induce HSPC proliferation and differentiation, or alternatively, (ii) the alterations could be caused by indirect pathogen sensing via mature hematopoietic and nonhematopoietic cells. These possibilities are not mutually exclusive, and both of them may involve PRR recognition of the pathogen.

To investigate the possible direct interaction of MAMPs and TLRs on HSPCs, we previously developed a model of HSPC transplantation to demonstrate that HSPCs are directly stimulated by TLR2, TLR4, and TLR9 soluble agonists *in vivo* and that the engagement of these receptors induces differentiation toward macrophages (13). In this work, we used a similar *in vivo* model of HSPC transplantation to investigate the possible direct interaction of β-glucans (depleted zymosan) and dectin-1 on HSPCs. Using this model, we excluded any indirect effects (cytokines or soluble mediators released by surrounding cells) by administering depleted zymosan to dectin-1^−/−^ mice (CD45.2^+^) transplanted with HSPCs from the bone marrow of B6Ly5.1 mice (CD45.1^+^). Moreover, the recipient mice were not irradiated in order to avoid an inflammatory environment that may generate artifact results. In accordance with our previous results (13, 22) and with Massberg et al. (23), who showed that migratory HSPCs give rise to myeloid cells in peripheral tissues, transplanted HSPCs in the absence of a dectin-1 challenge lose stem cell markers and differentiate into the myeloid lineage. However, the injection of depleted zymosan into the transplanted dectin-1^−/−^ mice significantly increases the yield of transplanted cells as well as the percentage of macrophages. These results demonstrate that production of myeloid cells by β-glucan administration *in vivo* involves a direct ligand-dectin-1 interaction on HSPCs.

We then delved into the role of dectin-1 and TLR signaling in HSPC differentiation *in vivo* in response to the fungal pathogen C. albicans. To study this, bone marrow HSPCs from C57BL/6, dectin-1^−/−^, or MyD88^−/−^ mice (CD45.2^+^) were transplanted into B6Ly5.1 mice (CD45.1^+^), which were then injected with inactivated yeasts. C. albicans yeasts induced the production of significant numbers of CD45.2 macrophages in a dectin-1- and MyD88-dependent manner. It should be noted that although recipient cells can recognize and respond to yeasts, the differences in macrophage differentiation observed by dectin-1^−/−^ or MyD88^−/−^ donor cells, in comparison with that by control donor cells, may only be due to a defective dectin-1 or TLR signaling in transplanted progenitors, as indirect signals induced by host cells should be equal. These *in vivo* results correlate with our previous *in vitro* observations, in which C. albicans yeasts induce the differentiation of HSPCs toward the myeloid lineage through TLR2 and dectin-1 signaling (7–9). Therefore, our results show that HSPCs sense C. albicans *in vivo* and are directed to produce macrophages in a dectin-1- and TLR-dependent manner, although the role of TLRs seems to be more significant *in vivo*. Overall, these results strengthen the hypothesis that during an infection, MAMPs may directly induce HSPC proliferation and differentiation, thus contributing to emergency myelopoiesis.

Recent studies have challenged the dogma that immune memory is an exclusive characteristic of adaptive immunity. That is, innate immune cells (especially monocytes and macrophages) can display some memory characteristics and respond in a different way upon reexposure to the same or heterologous stimuli (24). For example, exposure of monocytes and macrophages to C. albicans enhances their subsequent response to stimulation (trained immunity) (25). In this context, our group has demonstrated that this concept of “innate immune memory” could be applied not only to mature myeloid cells but also to HSPCs. Using an *in vitro* differentiation model of HSPCs, we have demonstrated that exposure of HSPCs to C. albicans results in the production of trained macrophages, with a greater capacity to produce cytokines and higher fungicidal activity than control macrophages differentiated with M-CSF (11). In this work, using the HSPC transplantation model in which the recipient mice are unable to respond to depleted zymosan, we have demonstrated that dectin-1-stimulated HSPCs *in vivo* are subsequently directed to produce trained macrophages by a cell-autonomous mechanism. Therefore, this is the first experimental evidence *in vivo* showing that trained immunity induced by dectin-1 signaling takes place also in HSPCs, upstream in the hematopoietic system. Our results are in agreement with a previous report showing that intravenous vaccination with Mycobacterium bovis bacillus Calmette-Guérin “educates” HSCs to generate trained monocytes/macrophages that protect the mice against tuberculosis (26), supporting the idea that microorganisms direct HSPCs to generate better prepared myeloid cells to fight against an infection. Further studies will be necessary to directly demonstrate a role for dectin-1 in training HSPCs for protective responses against systemic candidiasis.

Once demonstrated that signaling via dectin-1 in HSPCs induces myeloid differentiation to trained macrophages *in vivo* in a cell-autonomous way, we set out to elucidate whether the mechanisms involved are cell intrinsic (direct) or due to autocrine/paracrine (indirect) effects. Here, using mixed (WT and KO) HSPC cocultures *in vitro*, we show that dectin-1-mediated differentiation to trained macrophages is mostly produced by indirect mechanisms, in response to both depleted zymosan and inactivated yeasts of C. albicans. Supporting the role of indirect mechanisms, it has been reported that signaling via TLRs in HSPCs makes them release copious amounts of cytokines that act in an autocrine/paracrine manner to induce myeloid differentiation (15, 27). On this matter, our group has described that the secretomes (conditioned media) of HSPCs in response to TLR2 ligands and C. albicans are able to induce myeloid differentiation of HSPCs (28). However, other authors have described that TLR2, TLR4, and TLR7 ligands are capable of directly inducing myeloid differentiation intrinsically (16–19). Myeloid differentiation of common myeloid progenitors can be directly induced by TLR7 signaling, which acts synergistically with type I interferons (17). Moreover, using a model of Pam_3_CSK_4_ or LPS injection in chimeric mice, TLR2^−/−^ or TLR4^−/−^ recipients transplanted with WT+ TLR2^−/−^ or WT+ TLR4^−/−^ bone marrow, respectively, have a greater response of WT HSCs than of KO HSCs, revealing that, indeed, HSCs directly sense TLR ligands. In agreement with the involvement of indirect pathways, KO HSCs were not fully impaired in their capacity to respond to TLR ligands (18, 19). Accordingly, our coculture experiments reveal that macrophage differentiation can be directly induced by TLR2 signaling besides the indirect production of differentiating factors. Thus, understanding how this signaling pathway downstream of TLR2 regulates myeloid development in HSPCs requires further study. Moreover, future studies will be necessary to identify the molecules responsible for the secretome functions.

Finally, we also developed an *in vitro* experiment to approach as close as possible the conditions under which HSPCs encounter live cells of C. albicans during a real infection, once the fungus reaches the bone marrow (9) or HSPCs are mobilized to the site of infection, as has been described in different bacterial infection models (29, 30). Interestingly, we found that a short transient exposure of HSPCs to live C. albicans cells prior to differentiation is sufficient to program a trained response of the macrophages subsequently derived from them using M-CSF. The trained phenotype of the macrophages was partially dependent on the recognition of C. albicans by both TLR2 and dectin-1 receptors on HSPCs and was different for each specific cytokine. Trained TNF-α production was totally dependent on both TLR2 and dectin-1; trained IL-6 production was partially dependent on both TLR2 and dectin-1, whereas MIP-2 production was dependent on dectin-1 but not on TLR2. It is likely that compensatory signaling via remaining PRRs may be responsible for a lack of phenotype or an attenuated trained response. Overall, these data reinforce a novel mechanism whereby macrophage responses can be programed by PRR signaling in HSPCs prior to differentiation.

In conclusion, dectin-1 ligation activates HSPCs *in vivo* and induces their differentiation to trained macrophages by a cell-autonomous indirect mechanism. This points to a new mechanism by which β-glucans from fungal pathogens may modulate hematopoiesis in real time to generate myeloid cells better prepared to deal with the infection. Our results strongly support the role of dectin-1 in training HSPCs for a protective response against infection, although the direct demonstration will require further studies. Manipulation of this process may help improve the innate immune response during serious infections.

## MATERIALS AND METHODS

### Mice.

Dectin-1^−/−^ and MyD88^−/−^ mice were purchased from The Jackson Laboratory; TLR2^−/−^ were provided by Shizuo Akira (Osaka University, Osaka, Japan). All knockout mice have a C57BL/6 background and were maintained at the animal production service facilities (SCSIE, University of Valencia). Wild-type C57BL/6 mice (Envigo) were used as controls; wild-type CD45.1-positive allotype mice (B6.SJL-*Ptprc^a^ Pepc^b^*/BoyCrl strain, also known as C57BL/6-Ly5.1 [Charles River Laboratories]) and the transgenic mice (B6.Cg-Tg[CAG-DsRed*MST]1Nagy/J strain, also known as DsRed.T3 [The Jackson Laboratory]) were used for *in vivo* transplantation assays and/or for the *in vitro* coculture assays. Experiments were conducted with 8- to 12-week-old mice (regardless of sex). Experiments were approved by the Committee on the Ethics of Animal Experiments of the University of Valencia (permit numbers 2017/VSC/PEA/00207 and 2019/VSC/PEA/0030) and performed according to Spanish law under Reales Decretos 1201/2005 and 53/2013. All efforts were made to minimize suffering.

### Purification of Lin^−^ cells.

Mouse HSPCs were isolated as lineage-marker-negative cells (Lin**^−^** cells) from bone marrow. Briefly, murine bone marrow was obtained by flushing the femurs and tibias; cells were depleted of lineage-positive cells by immunomagnetic cell sorting using MicroBeads (Miltenyi Biotec): bone marrow cells were labeled with a cocktail of antibodies against a panel of lineage antigens (CD5, CD45R [B220], CD11b, Gr-1 [Ly-6G/C], 7-4, and Ter-119), and then cells were purified by negative selection according to the manufacturer’s instructions. Purity of the sorted cells was assessed by labeling with anti-Lin cocktail and by flow cytometry analysis, and no Lin^+^ cells were detected.

### PAMPs and preparation of fungal stimuli.

The stimuli used were depleted zymosan, Pam_3_CSK_4_, ultrapure Escherichia coli LPS (all from Invivogen), or inactivated C. albicans ATCC 26555 yeasts obtained as previously reported (12, 28, 31). Briefly, yeasts were grown in YPD medium (1% yeast extract, 2% peptone, 2% glucose) at 28°C up to the late exponential growth phase (*A*_600_ of 0.6 to 1), collected, and washed with water. Cells were resuspended in water and maintained for 3 h at 28°C with shaking and afterwards at 4°C for 24 h (starved yeast cells). Starved yeast cells were inoculated (200 μg [dry weight] of cells per ml) in a minimal synthetic medium and incubated for 3 h at 28°C. For inactivation, yeast cells were resuspended (20 × 10^6^ cells/ml) in BD Cytofix fixation buffer and incubated for 1 h at room temperature. After treatment, fungal cells were extensively washed in phosphate-buffered saline (PBS) and brought to the desired cell density in cell culture medium. Viable yeasts for *in vitro* assays were grown in YPD medium at 28°C up to the late exponential growth phase, collected, washed in PBS, and brought to the desired cell density in cell culture medium. All procedures were performed under conditions designed to minimize endotoxin contamination as described elsewhere (31).

### *In vivo* transplantation and stimulation of Lin^−^ cells.

Approximately 2.5 × 10^6^ Lin**^−^** cells in 100 μl of PBS (purified from four mice of the designated strain) were intravenously injected into one recipient mouse (of the other designated strain). Transplanted mice were then injected with PBS (control) or with different stimuli: depleted zymosan (300 μg), inactivated yeasts (10^7^ cells), macrophage colony-stimulating factor (M-CSF; 10 μg [Miltenyi Biotech]), or M-CSF and depleted zymosan, once daily for 3 days.

### Detection and characterization of transplanted cells.

In the transplantation model that takes advantage of the polymorphisms of CD45 (CD45.1 and CD45.2 alleles) for *in vivo* tracking, the procedure was as follows. Each transplanted mouse was killed at day three, and the spleens and bone marrow from femurs and tibias were removed aseptically. Total spleen cells were obtained by collagenase D treatment of the organ as previously described (32, 33). Erythrocytes were lysed in BD FACS lysing solution (BD Bioscience). Fc receptors were blocked with FcR blocking reagent (Miltenyi Biotec), and the sample was depleted of cells that express the CD45 alloantigen of the recipient mice (in order to enrich the sample for the transplanted cells) by immunomagnetic cell purification using biotinylated anti-CD45.1 or -CD45.2 antibody and anti-biotin magnetic MicroBeads (both from Miltenyi Biotec). Recovered cells were microscopically counted, labeled with various combinations of antibodies, and analyzed by flow cytometry (see below).

In the transplantation model that takes advantage of the DsRed expression for *in vivo* tracking, mice were injected with M-CSF or M-CSF and depleted zymosan (as described above); mice were then sacrificed and their spleens were removed. Total spleen cells were obtained as described above, and the sample was depleted of T and B cells (in order to enrich the sample for myeloid cells) by immunomagnetic cell purification using biotinylated anti-CD3 and anti-CD19 antibodies and anti-biotin magnetic MicroBeads (all from Miltenyi Biotec). The remaining cells were stained for CD11b and F4/80, and DsRed double-positive cells were sorted by flow cytometry using a FACSAria Fusion cell sorter (BD Bioscience), plated, and stimulated the following day for the analysis of cytokine production.

### *In vitro* culture experiments.

Purified Lin**^−^** cells were immediately cultured in complete cell culture medium (RPMI 1640 medium supplemented with 2 mM l-glutamine, 5% heat-inactivated fetal bovine serum, and 1% penicillin-streptomycin stock solution [Gibco]) supplemented with 20 ng/ml stem cell factor (SCF; Peprotech). Where indicated, cultures also contained 50 ng/ml M-CSF to induce differentiation to macrophages and/or PRR agonists or C. albicans cells as stimuli. At different days of coculture, cells were microscopically counted and (i) labeled with various combinations of antibodies and analyzed by flow cytometry (see below) or (ii) sorted to separately plate DsRed-positive and -negative macrophages to stimulate them the following day to asses cytokine production.

### Transwell assays.

Transwell assays were performed using 12-well plates with 0.4-μm-pore polycarbonate membranes from Corning. Lin**^−^** cells were plated in the upper and lower chambers (50,000 cells in each chamber), and macrophages were derived from them using M-CSF for 7 days. Where indicated, lower chamber cultures also contained depleted zymosan or inactivated C. albicans cells as stimuli. Adherent macrophages were harvested from the lower and upper chambers on day 7, plated at equal numbers, and stimulated for the analysis of cytokine production.

### Measurement of cytokine production.

Macrophages were plated in flat-bottomed 96-well plates at a density of 50,000 cells in 200 μl of complete cell culture medium. Cells were challenged with the indicated stimuli for 24 h, and cell-free supernatants were then harvested and tested for cytokine release using commercial enzyme-linked immunosorbent assay (ELISA) kits (TNF-α and IL-6 [eBioscience]; MIP-2 [R&D Systems]) Unstimulated macrophages served as negative controls. Triplicate samples were analyzed in each assay.

### Antibodies and flow cytometry analyses.

Cell suspensions were labeled with various combinations of antibodies and analyzed by flow cytometry. The following antibodies were used in this study: phycoerythrin (PE)-labeled anti-CD11b (clone M1/70 from eBioscience), fluorescein isothiocyanate (FITC)-labeled anti-CD11b (clone M1/70 from eBioscience), allophycocyanin (APC)-labeled anti-F4/80 (clone BM8 from eBioscience), PE-Cy7-labeled anti-F4/80 (clone BM8 from eBioscience), FITC-labeled anti-CD45.1 (clone A20 from Miltenyi Biotec), and FITC-labeled anti-CD45.2 (clone 104-2 from Miltenyi Biotec). Flow cytometry analyses were performed on an LSR Fortessa cytometer (BD Biosciences), and data were analyzed with FACSDiva and FlowJo 10 software.

### Statistical analysis.

Statistical differences were determined using one-way analysis of variance (ANOVA) followed by Dunnett’s test for multiple comparisons and two-tailed Student's *t* test for dual comparisons. Data are expressed as means ± standard deviations (SDs). Significance was accepted at a *P* value of *<*0.05.
